# Machine Vision-Based Fatigue Crack Propagation System †

**DOI:** 10.3390/s22186852

**Published:** 2022-09-10

**Authors:** Jan Gebauer, Pavel Šofer, Martin Jurek, Renata Wagnerová, Jiří Czebe

**Affiliations:** Department of Control Systems and Instrumentation, VŠB—Technical University of Ostrava, 708 00 Ostrava, Czech Republic

**Keywords:** crack, propagation, surface crack, machine vision, National Instruments, Vision Builder

## Abstract

This paper introduces a machine vision-based system promising low-cost solution for detecting a fatigue crack propagation caused by alternating mechanical stresses. The fatigue crack in technical components usually starts on surfaces at stress concentration points. The presented system was designed to substitute a strain gauge sensor-based measurement using an industrial camera in cooperation with branding software. This paper presents implementation of a machine vision system and algorithm outputs taking on fatigue crack propagation samples.

## 1. Introduction

The presented publication deals with replacement of a strain gauge sensor by a machine-vision solution. The target of the research was to replace the actual strain gauge method in train wheel testing stand with a new more reliable and easier to operate method.

The train wheelset manufacturer must guarantee to its customers that the required properties of train wheel unit will last throughout the service life. The lifetime of train wheels and their axles is in the order of millions of kilometres. The train wheels are designed regarding several parameters, including the type of locomotive or the expected load. An important role in the design of the train wheels is also played by the climate prevailing in the country for which the gearing is intended. The designed shape and exact material composition must withstand all of these requirements for the lifetime of the whole train wheels unit.

**Figure 1:**
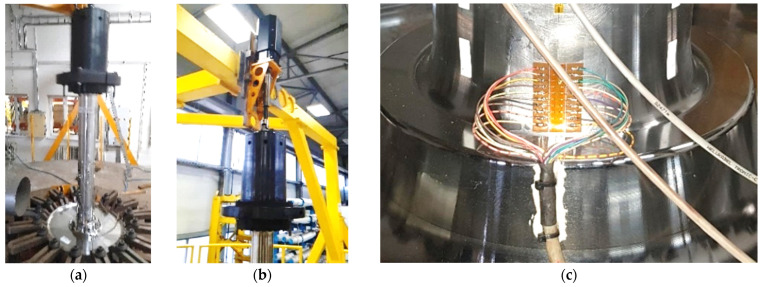
(**a**) The testing platform for a train wheel with axles. (**b**) Detail of an actuator with universal joint. (**c**) Detail of a strain gauge array placed over artificial notch on the tested specimen.

Before a mass production starts, testing of the first pieces must take place. Indirect measurement of crack size using a strain gauge array was used in testing (Figure 1). The strain gauge measurement is one part of whole testing stand. The output of strain gauge measurement is information about the current length of the crack propagating on the artificial notch. The parameters of test (amplitude, frequency, and load) are commonly changed in time, depending on the length of crack. The specimen test result is the number of cycles to desired crack length and number of cycles to specimen destruction. The accurate crack length information is crucial for changing the testing parameters setup during specimen testing. The measurement of length with strain gauges was unsuitable for its inacceptable accuracy and infinitesimal repeatability.

This article describes the testing of machine-based crack measurement on a laboratory testing stand. The specimen used for laboratory testing was created from material provided by a train wheels manufacturer. For an algorithm design of the crack measurement, it was important that the optical surface properties of laboratory specimen were the same as the original train wheels unit, primarily the colour, reflectivity, and surface roughness.

Machine-vision based measurement is a contactless method of specimen probing. This method of measurement (unlike strain gauges) does not need any additional material, it is easily portable to another type of specimen (in both laboratory and industrial environments). The strain gauge measurement requires a new set of sensors for each measurement, including precise gluing and wire soldering.

With sufficiently high-quality lighting and high resolution of today’s cameras, the presented method is sufficiently accurate and achieves high repeatability for usage in industrial applications.

The described crack measurement system is focused on fatigue crack length measurement on specimen under constant amplitude loading. Unlike slow tensile tests or fast impact tests, fatigue is based on cyclic loading of specimen. Fatigue is the general phenomenon of material failure after several cycles of loading below the tensile strength [1].

The measurement parameters, such as S-N (stress–number of cycles) diagrams, are defined according to the actual testing needs. The load changes with the number of cycles or time.

**Figure 2:**
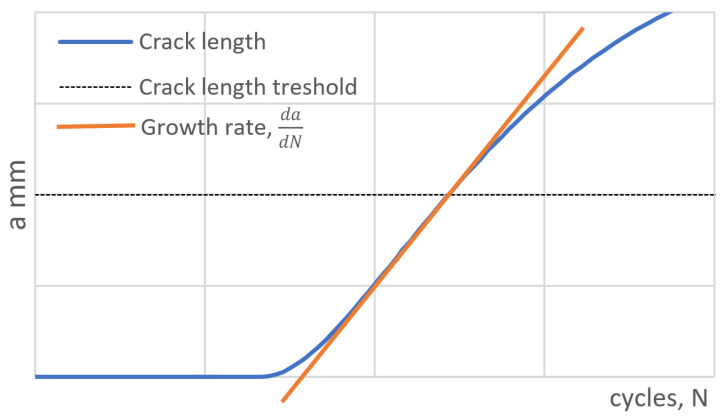
Growth length rate illustration (values of stress and cycles are not in scale).

The working procedure of specimen testing has defined specific requirements for the shape of the S-N curve, changing with the length of the crack. In these cases, the length of the crack needs to be measured periodically during the test. Given stress level relates to the crack length or its growth rate (Figure 2).

In this approach the number of cycles is varying based on specimen and testing profile properties (stress level, time, frequency, and stress pattern).

Various sensing approaches were studied for detecting and monitoring fatigue cracks in structures [2,3,4,5]. The basic method is a direct and indirect visual inspection. Direct visual inspection is usually performed by eye, magnifying glass, or microscope. For indirect visual inspection, optical instruments such as an endoscope or machine vision are used [6]. Ultrasound is often used to detect subsurface defects in materials. Using an ultrasonic probe, an ultrasonic pulse is cyclically transmitted to the examined object. When in the case of the existence of a subsurface defect, a part of the pulse is reflected from this defect and is detected back as a partial echo [7,8,9]. A very effective method is an acoustic emission [10,11,12,13]. This method detects acoustic signals in the tested object, which are generated by the sudden release of energy in the material, mostly by initiation and propagation of a crack in the structure. By using a network of very sensitive acoustic probes, an initiated or propagating crack in the material can not only be detected but also localized. The disadvantage of the method is its extreme sensitivity to external acoustic interference. This interference can be generated by friction between components of the structure under test, which generates acoustic signals in which the detected echo can be lost.

The mentioned NDT methods are used for stationary applications, where the used hardware, including the sensitive analyser, are not in motion or loaded by vibrations. These include inspection of welds, inspection of pressure vessels, piping, or structural health. The other methods—such as application of carbon nanotubes (CNT) layer [14], strain gauge sensing [15,16], vision-based inspection of concrete structures [17,18] where convolutional methods are mostly used [19,20,21], or big data analysis [22]—are not considerable under vibrations affected surfaces and environments.

In industrial application, which we focused on train wheel testing in Figure 2, The camera must be mounted directly on the stressed wheel (up to 20 Hz, 95 G). This brings challenging conditions for used testing hardware.

The target of presented vision-based measurement is to replace the currently used strain gauge array sensor detecting the fatigue crack. Reliability of this approach depends on the quality of how the strain gauge is linked to the surface. Our system is designed to replace this method in a more precise and reliable way.

The presented publication describes an application where it was necessary to detect the initiation and progression of a fatigue crack from an artificial notch on a specimen. A similar problem was solved [23,24,25] using convolutional networks in our previous paper [26].

**Figure 3:**
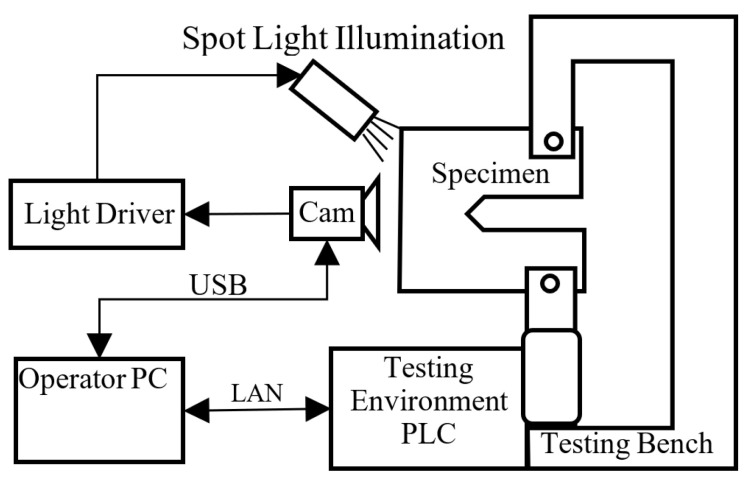
Simplified scheme of vision system components connection in testing environment.

**Figure 4:**
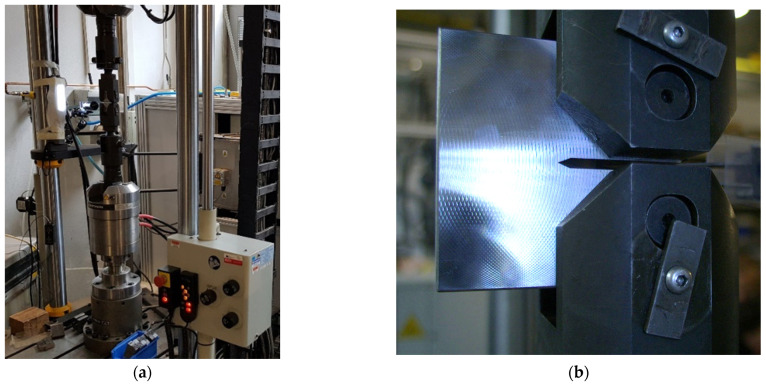
(**a**) The vision system implemented in industrial setup. (**b**) Detail of the specimen arrangement in the testing stand.

The tested sample was cyclically stressed in the test equipment, (see Figure 3 and Figure 4) and after a certain number of cycles, a fatigue crack began to propagate from the notch. The task was to capture the moment of formation of this crack and, at the same time, measure its size in real time. Despite initial experiments, mentioned NDT methods proved to be unsuitable. Due to the large vibrations, the use of real-time optical image evaluation proved to be optimal.

## 2. Industrial Integration

An industrial environment provides challenging conditions, including heat, dust, and (in our case) most of all, vibrations. For algorithm and hardware setup, a small stand for dynamic material testing was used [24,25]. Testing parameters and setup were chosen to fit industrial standards for train wheels testing. Used dynamic force up to 250 kN, frequency up to 250 Hz and maximal acceleration up to 70 G were used (see Figure 4).

**Figure 5:**
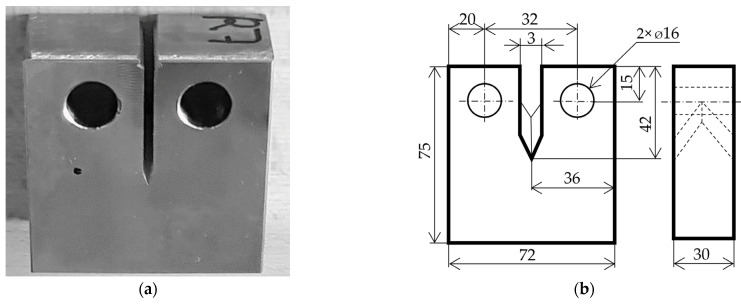
(**a**) Detail of specimen with artificial notch on surface. (**b**) Drawing of specimen with fundamental dimensions in millimetres.

Selected specimen material is used in train wheelsets (see Figure 5). The material manufacturers code is R7, with following mechanical properties: proof strength (0.2%)—547 MPa, tensile strength—860 Mpa, average hardness—2.68 Gpa, and total elongation >26%. The chemical compositions of the steels are given as percentages by weight (%wt): C—0.541, Si—0.268, Mn—0.772, Cr—0.238, and Ni—0.149. The testing hardware parameters setup depends on each individual application.

In the presented setup, the camera is mounted directly to the frame of the testing bench, which is mounted on solid surface. The whole test of specimen takes more than 5 h until the fatigue crack is initiated, and more than 5 h until the tested sample is destroyed. In this case, the frame rate of the camera is 1 frame per 10 s.

## 3. System Hardware Description

All hardware parts must be ready to work for several days in a single run, despite dust of metal oxides and vibrations, the most dangerous condition of environment. This is very challenging for camera lenses, and the camera itself. We chose precise lenses (Kowa Optimed Deutschland GmbH, Dusseldorf, Germany, F1.4/16 mm) specifically for these conditions. The focus ring is fixed by tightening the ring, and aperture setting is determined by a set of changeable parts (iris) inside of lenses.

The camera used in our example (Basler Electric Company, Highland, IL, USA, acA2440-35uc) was also designed for rough conditions, and was equipped with USB 3 interface and output for lighting driver solution (Basler SLP Strobe Controller 121040). The Opto Engineering LTPRUP-W light and LTDV1CH-17V driver were used for lighting. Operators PC is placed out of testing hall. It is equipped by National Instruments Vision Builder SW with runtime license. This type of license allows the operator to change only algorithm parameters, not the algorithm itself. The presented solution is simpler for application than a solution with strain gauge array, which needs additional hardware and supplementary materials.

## 4. Lighting Setup

**Figure 6:**
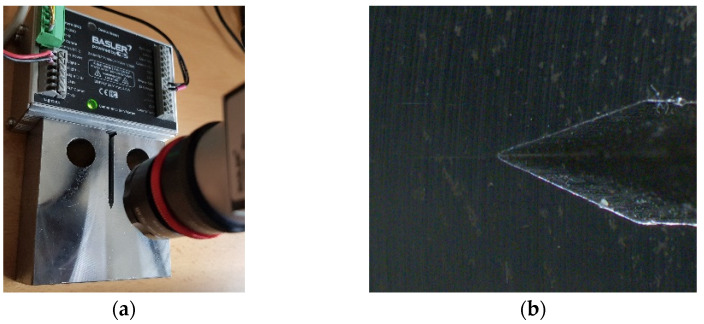
(**a**) The camera setup. (**b**) The image of area of interest with natural ambient illumination.

The process of selection of correct lighting components was essential for this application. Any precise industrial vision system cannot work properly with natural light conditions. Without appropriate and consistent illumination of captured scene the subject of our interest—fatigue crack—is unnoticeable even with the human eye (see Figure 6).

The target of any illuminator is to make the object of interest visible for subsequent machine vision processing. Figure 6, and others in this chapter, shows setup for illumination testing with an already grown crack on the specimen.

### 4.1. Dark Field Illumination

When illuminating reflective objects, there is a necessity to solve problems with unwanted reflections, in which the design of the image completely fades.

**Figure 7:**
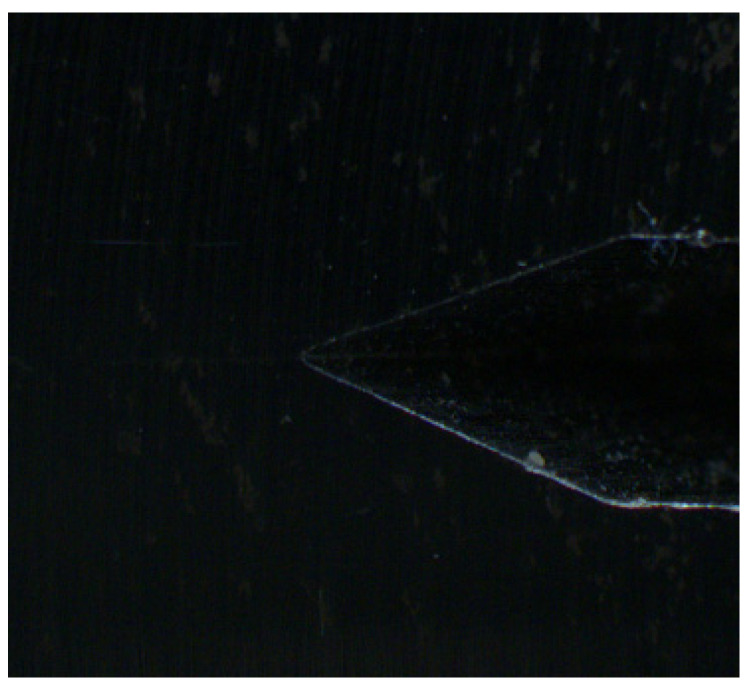
The image of area of interest with dark field illumination.

Dark field illumination is conducted by a flat ring light that is mounted very close to the object. The result is that most of the light is reflected away from the camera sensor. The image of the tested subject in Figure 7 shows the type of effect that can be obtained when using dark field lighting. This low angle lighting is ideal for edge detection and damage on metal surface. The gained image has the high purity and quality of expressing details. Regrettably, the area around the fatigue crack is completely dark. In our case, the crack is invisible due to its low profile under the surrounding surface.

### 4.2. Direct Circular Illumination

**Figure 8:**
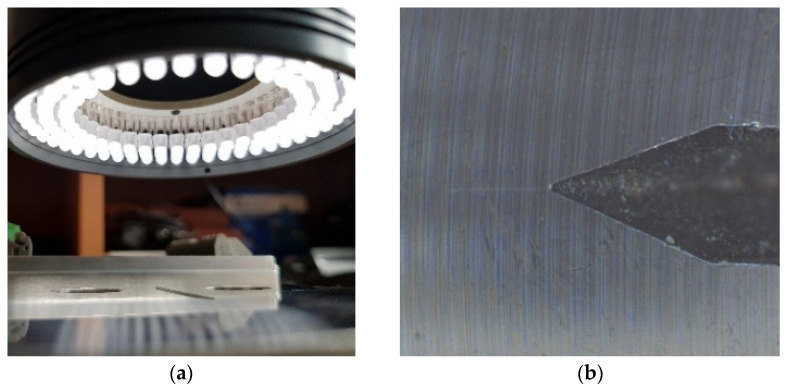
(**a**) The camera setup and lighting. (**b**) The image of area of interest with direct circular illumination.

The direct circular light illuminates the object directly from a flat board. It should be suitable to detect the smallest divergences of reflectance in the focused area (see Figure 8). On this lighting setup, the fatigue crack is slightly recognizable. Unfortunately, the contrast and colour profile of the crack is very similar to the surrounding of the crack and is hardly detectable by machine vision.

### 4.3. High Brightness Spot Light Illumination

**Figure 9:**
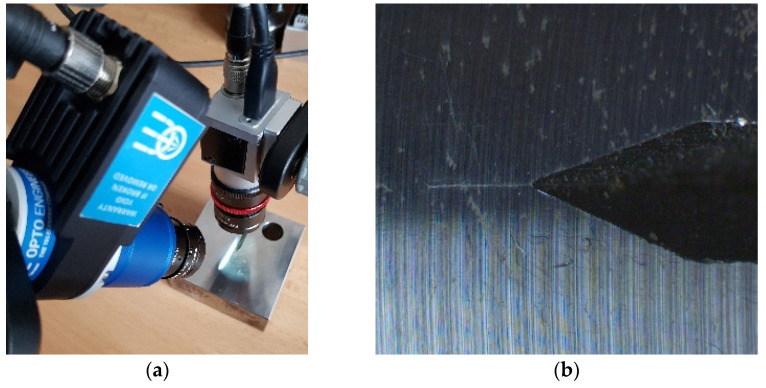
(**a**) The camera and lighting setup. (**b**) The image of area of interest with spot-light illumination.

The usage of spot-light solution emerged as the best option (see Figure 9). It was a high-power strobe spotlight (17A in peak) with a lens for maximum illuminance and long working distance.

On the captured image, there is a clearly observable crack on the dark background, which was our goal to make the fatigue crack as bright as possible. This lighting solution enables camera to use exposure time 1/10,000 of second. This very short exposure time avoids blurry images and enables the use of this technology, even if it is not placed directly on the tested object.

## 5. Fatigue Crack Detecting Algorithm

**Figure 10:**
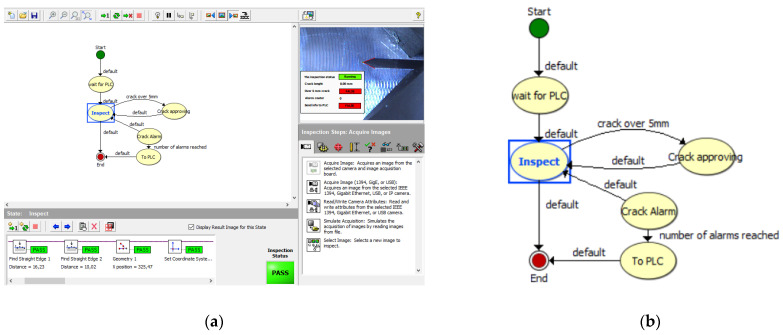
(**a**) Basic schemes of system states in the NI Vision Builder environment. (**b**) The detail of state flow diagram.

The whole crack detecting system is based on the National Instruments Vision Builder environment. The presented algorithm is based on the state flow scheme (see Figure 10).

As it was mentioned in the introduction, the whole process of crack propagation can take from several hours up to days. The operator of the testing environment is the responsible one for the whole test progress control. Our vision system is a part of the whole testing environment and in this way, it is designed to be easy to operate by the system operator. Once the operator runs application and fits parameters for actual testing, the algorithm is then fully automatic with no necessity of operator interaction. It fully cooperates with PLC, which controls the process of the testing environment.

### 5.1. Basic Inspection (Inspect State)

**Figure 11:**
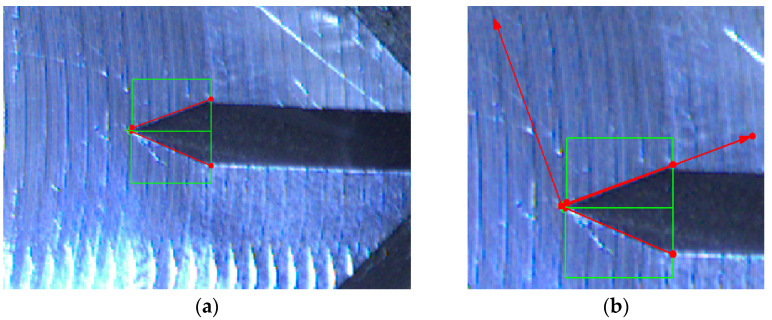
(**a**) The edge detection (green: detection zone, red: detected line). (**b**) The intersection finding (green: detection zone, red: detected line and coordinates position).

The first step is to create a steady point in picture to determinate the initial position of coordinate system for each frame. It is achieved by finding intersection of two lines, detected on edges of the detected subject (Figure 11).

The algorithms for edge detection are relatively straightforward. Edges can be characterized intuitively, in geometric terms, as the projection of object surface marks and other elements of an image.

The intersection of two edge boundaries provides a steady feature. Such features often prove to be stable across sequences of images, and are therefore useful in tracking objects across sequences.

**Figure 12:**
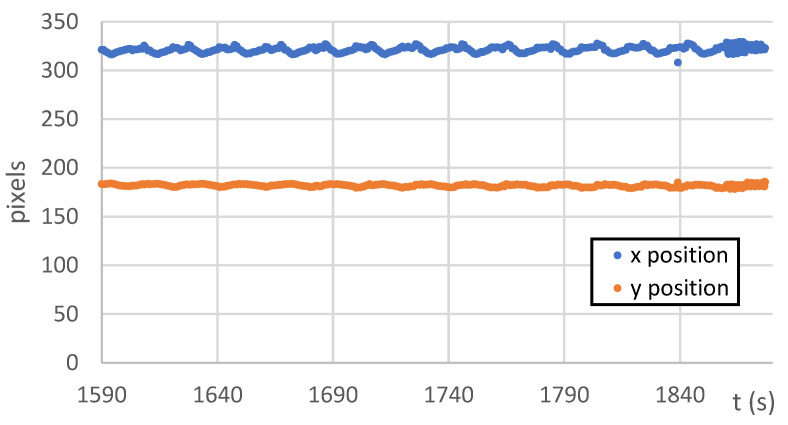
The artificial notch position over time.

**Figure 13:**
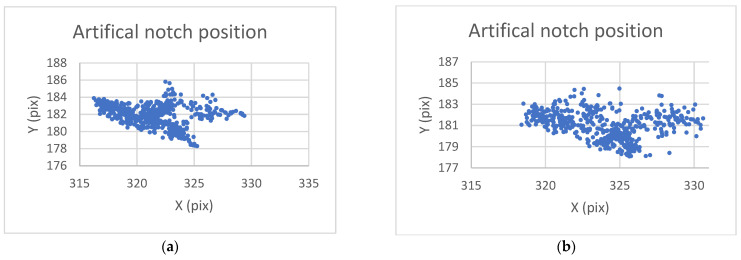
(**a**) The artificial notch position throughout the time of test (setup 1). (**b**) The artificial notch position throughout the time of test (setup 2).

The analysis of this step (Figure 12 and Figure 13) shows standard behaviour of artificial notch position on acquired frames under stress. This method of intersection finding is reliable and suitable for next processing.

Figure 13a,b shows the difference between possible line finding setups and its effect on tracking of artificial notch position. This setup must be prepared carefully before the test by the testing operator. Parameters of line finding depend on actual specimen and lighting conditions.

The next step is a determination of zones for the crack inspection. In our case, it is necessary to determinate the initiation time of fatigue crack propagation and length of the crack. The presented approach offers enhanced 0.5 mm resolution, instead of 5 mm resolution used in our subsequent work [26]. This allows us to determine the growth rate of the crack.

**Figure 14:**
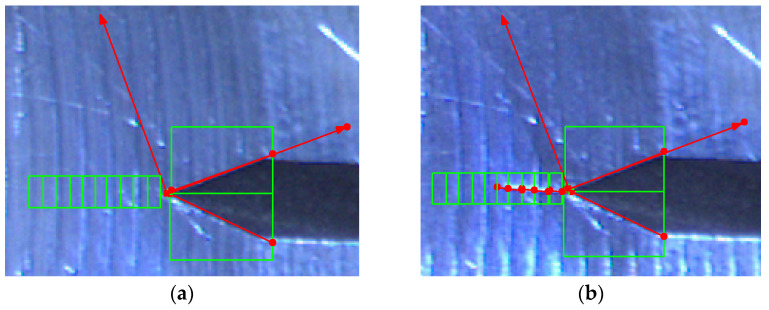
(**a**) Selection of line search zones (green: detection zones, red: detected lines). (**b**) Detection of crack in segments (green: detection zones, red: detected lines).

Since the 0.5 mm resolution is sufficient for the detection system output, several 0.5 mm zones of straight-line finding are determined (see Figure 14). Output of this module is pass/fail information about the crack length in 0.5 mm accuracy. When the straight line of the desired length (setpoint) is detected, the system goes into the next state (“Crack approving”) to more detailed inspection of actual frame.

The system resolution depends on camera parameters and position on actual the specimen. In the presented example, resolution is 45 pixels/mm.

**Figure 15:**
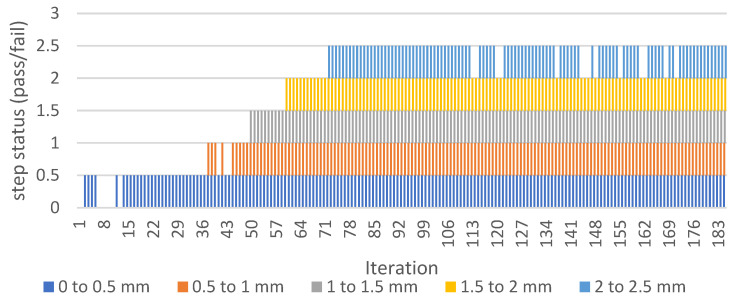
Find Straight Edge-Step Status for each line finding segment.

The calculated length of the crack is a sum of all line detection zone-segments. Figure 15 shows the output of all segments over time. It is observable that the system occasionally fails at the beginning of the crack development under the detection zone.

**Figure 16:**
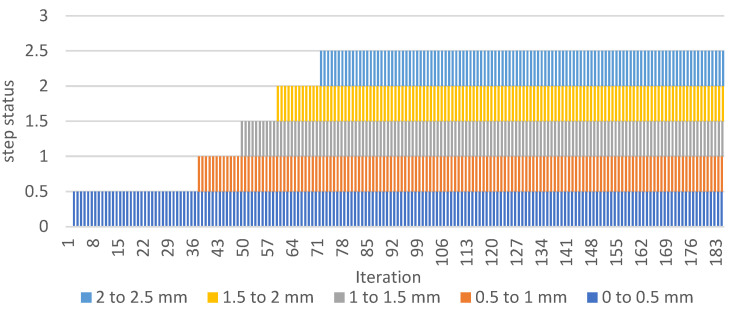
Find Straight Edge-Step Status (corrected).

This fail phenomenon is corrected by an additional step, where failure states are recognized on each detection zone (Figure 15) and replaced by nonzero value (Figure 16).

**Figure 17:**
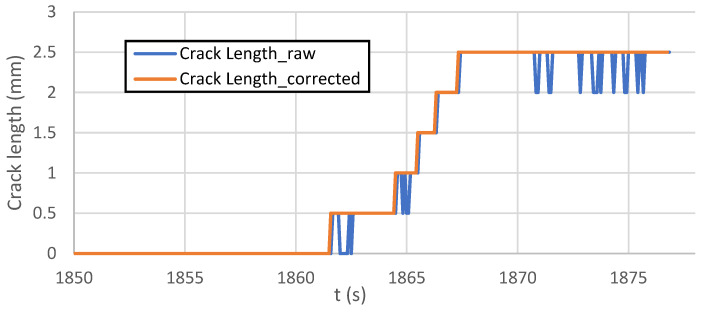
The graph of fatigue crack propagation over time.

Figure 17 shows the graph (envelope of values of previous graphs on Figure 15 and Figure 16) output and comparison of corrected/incorrected length output variable.

**Figure 18:**
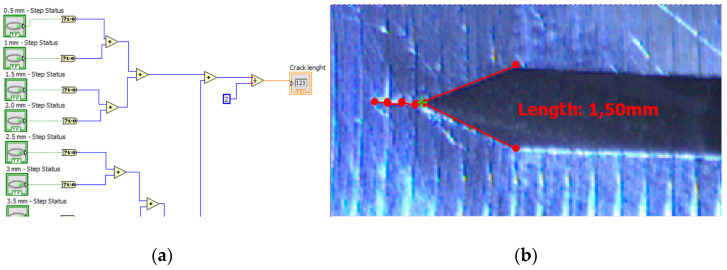
(**a**) The fragment of the logical operation behind crack length measurement. (**b**) Numerical interpretation of function “calculator” as custom overlay.

The presented graphs above are based on output of “Calculator” and “Array operator” functions in Figure 18. More detailed explanation of the line detection failure and its avoidance can be found in the next section.

### 5.2. Crack Confirmation (Crack Approving State)

**Figure 19:**
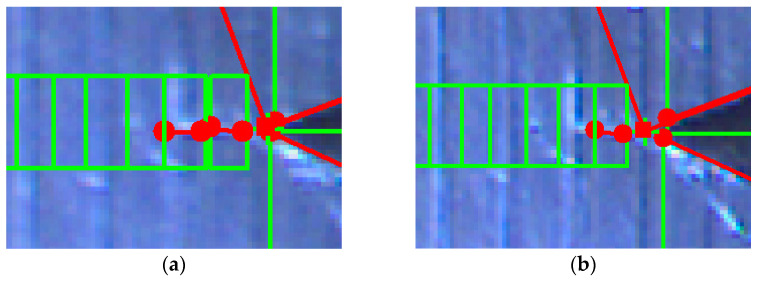
(**a**) The correctly detected line (green: detection zones, red: detected objects). (**b**) The incorrectly identified straight line (green: detection zones, red: detected objects).

The detection of a crack using “find straight edge” function in previous parts of the algorithm is occasionally incorrect (Figure 17 and Figure 19). To eliminate mistakes in a crack measurement, more detailed inspection follows.

The crack confirmation process starts once the measured length of a crack reaches the setpoint value (in our case, 5 mm).

The confirmation of a crack is based on matching of geometry by templates. This method brings an accurate output of position and matching score of found objects. In our case, founded pattern match means a mistakenly detected crack. If some matches are found with prepared pattern mask, the actual frame inspection is ended without alarm indexing. If there is no match with pattern, the alarm counter variable is increased by one and the algorithm proceeds to another image.

**Figure 20:**
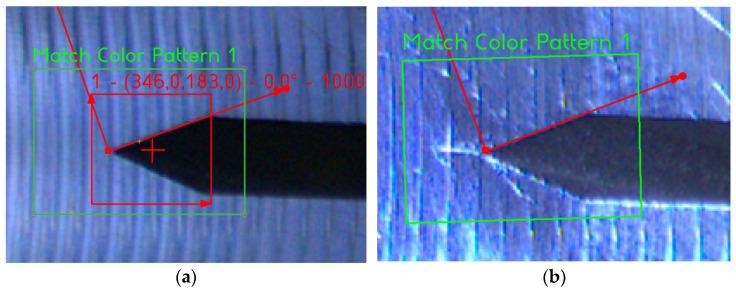
(**a**) The pattern matching input. (**b**) The correctly approved crack.

**Figure 21:**
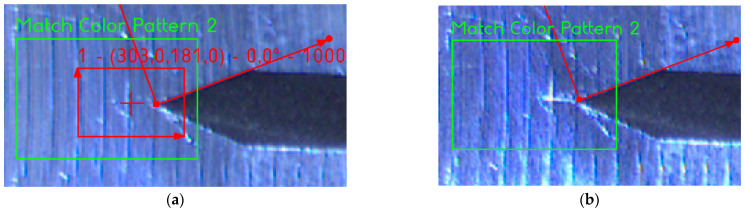
(**a**) The pattern matching input. (**b**) The correctly approved crack.

Figure 20a and Figure 21a show two initial patterns (red square) in the region of interest (green square). Once the crack is distinguishable, the “no match” output is signalised (see Figure 20b and Figure 21b).

**Figure 22:**
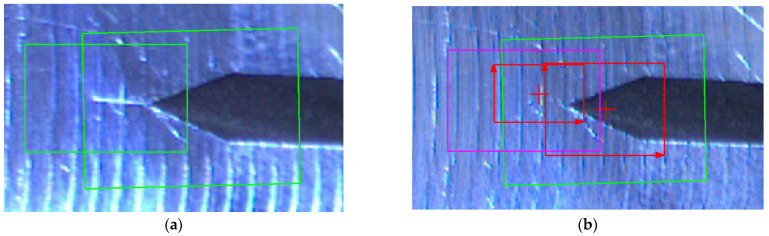
(**a**) No template match found in correctly marked frame for detailed inspection (green: detection zones). (**b**) Template match found in incorrectly marked frame for detailed inspection (green and pink: detection zones, red: detected patterns).

Figure 22a shows no matches found on the actual frame. In this case, the algorithm continues back to the inspection state. Figure 22b shows two matches found on the suspect frame. In this case, the algorithm increases the alarm counter by one.

The output of the “Crack approving” state is then controlled by the “alarm counter” variable. If the number of alarms is higher than the desired value (depending on actual industrial application setup), the algorithm goes into the next state (“To PLC”) to inform higher level controller (PLC) of testing environment, or goes back to “inspect” state and continues with the inspection.

### 5.3. Crack Alarm State

The last state of the algorithm ensures communication between PC and higher-level PLC which controls testing parameters and conditions of testing stand.

Communication is linked up by ethernet using TCP assembly to read and write data using TCP I/O function. When a crack is detected and approved by the algorithm, there are two types of algorithm outputs: first is sending of the data package to higher level PLC; second is overlaying screen information for the operator.

Alarm counter setup is one parameter set by the operator before the test starts. The value of alarm count depends on the operator experience and testing conditions.

### 5.4. Overlay Information Panel

**Figure 23:**
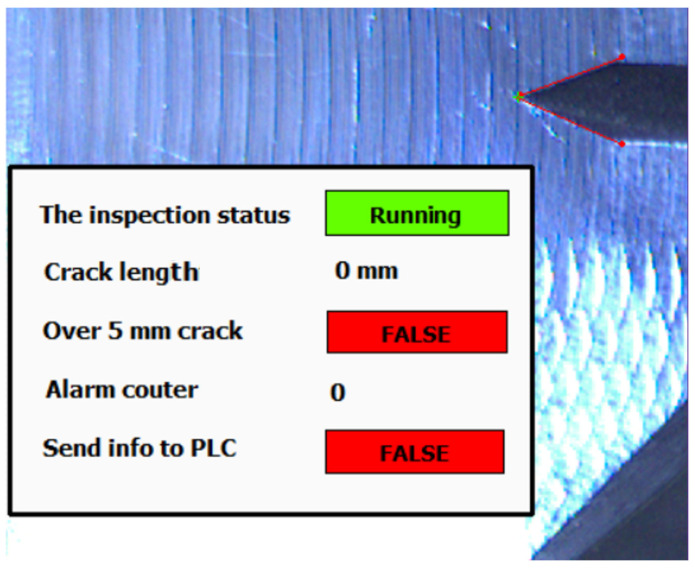
The overlay panel in inspection application.

**Figure 24:**
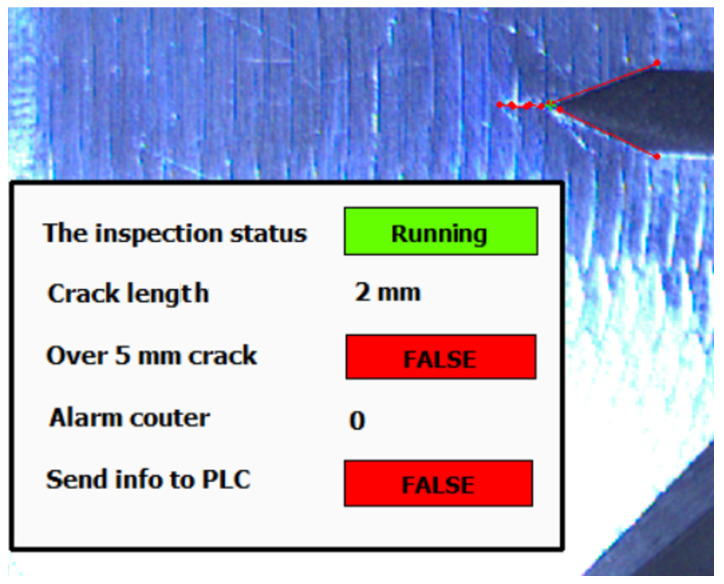
The overlay panel in inspection application.

The testing environment operator can constantly monitor progress of the tested subject by provided application. The user overlay provides live information about inspection status (see Figure 23 and Figure 24).

The operator can set up parameters and observe an actual acquired image with few status indicators. The most important parameter for the operator is the setpoint indicator “Over 5 mm crack”. This is information of a breakpoint of the whole testing procedure. The alarm is activated when the crack length setpoint limit is reached. This value could be changed before the test starts.

When the crack alarm occurs and is approved, the indicated frame is sent to the operator PC in an archive folder with log file for forthcoming manual inspection. Based on alarm counter setup, the number of saved images is between 3 and 6. If the desired number of alarms is reached, the algorithm sends this status to PLC.

After this event, the mode of the testing environment is automatically changed and continues with different parameters (lower frequency of oscillation, lower amplitude) until the specimen is destroyed. This final mode of testing does not need any machine inspection.

## 6. Conclusions

This paper describes the application of a real-time machine vision-based measurement system applied in an industrial environment, specifically the railway industry. It presents a reliable approach to real-time measurement of fatigue cracks on metal surface in rough industrial conditions. The used hardware successfully passed in unfavourable environments including a high level of heat, dust and mainly vibrations.

The algorithm presented in this article was created in the NI Vision Builder environment. A build-in function block enables basic image inspection. Above all, the presented solution brought quick and satisfactory results. In industry, it was possible to deploy this system after a few weeks from the assignment. A surface crack appears at the point of energy concentration. This point is not in a permanent place of acquired picture, due to vibrations. It was necessary to trace the change of position and adjust the position of the coordinate system for each acquired image.

If the crack is detected, the image is analysed a second time using a mask comparison. If the crack of required length is confirmed, it will be recorded as an alarm event. If the limit of alarm messages is exceeded, the operator and higher-level PLC of system control are informed. The operator has an option to perform a manual visual inspection of suspicious frames.

Each industrial application and tested wheel unit is different (depending on actual wheel unit production). The operator must be trained and able to change the properties of some functions of the algorithm to work correctly. This provides creation of patterns to match and perform an initial setup of coordinates. The presented system allows this while basic runtime license of software is used.

## Data Availability

Not applicable.
